# Design of a randomized controlled trial for multiple cancer risk behaviors among Spanish-speaking Mexican-origin smokers

**DOI:** 10.1186/1471-2458-13-237

**Published:** 2013-03-18

**Authors:** Yessenia Castro, Karen Basen-Engquist, Maria E Fernandez, Larkin L Strong, Elizabeth G Eakin, Ken Resnicow, Yisheng Li, David W Wetter

**Affiliations:** 1 School of Social Work, The University of Texas at Austin, Austin, TX, USA; 2 Department of Behavioral Science, The University of Texas MD Anderson Cancer Center, Houston, TX, USA; 3 Division of Health Promotion and Behavioral Science, The University of Texas School of Public Health, Houston, TX, USA; 4 Department of Health Disparities Research, The University of Texas MD Anderson Cancer Center, Research - Unit 1440, PO Box 301402, Houston, TX, 77230-1402, USA; 5 Cancer Prevention Research Centre, School of Population Health, The University of Queensland, Brisbane, QLD, Australia; 6 Department of Health Behavior and Health Education, University of Michigan School of Public Health, Ann Arbor, MI, USA; 7 Department of Biostatistics, The University of Texas MD Anderson Cancer Center, Houston, TX, USA

**Keywords:** Latinos, Mexican, Mexican American, Smoking cessation, Fruits/vegetables, Physical activity

## Abstract

**Background:**

Smoking, poor diet, and physical inactivity account for as much as 60% of cancer risk. Latinos experience profound disparities in health behaviors, as well as the cancers associated with them. Currently, there is a dearth of controlled trials addressing these health behaviors among Latinos. Further, to the best of our knowledge, no studies address all three behaviors simultaneously, are culturally sensitive, and are guided by formative work with the target population. Latinos represent 14% of the U.S. population and are the fastest growing minority group in the country. Efforts to intervene on these important lifestyle factors among Latinos may accelerate the elimination of cancer-related health disparities.

**Methods/design:**

The proposed study will evaluate the efficacy of an evidence-based and theoretically-driven Motivation And Problem Solving (MAPS) intervention, adapted and culturally-tailored for reducing cancer risk related to smoking, poor diet, and physical inactivity among high-risk Mexican-origin smokers who are overweight/obese (n = 400). Participants will be randomly assigned to one of two groups: Health Education (HE) or MAPS (HE + up to 18 MAPS counseling calls over 18 months). Primary outcomes are smoking status, servings of fruits and vegetables, and both self-reported and objectively measured physical activity. Outcome assessments will occur at baseline, 6 months, 12 months, and 18 months.

**Discussion:**

The current study will contribute to a very limited evidence base on multiple risk factor intervention studies on Mexican-origin individuals and has the potential to inform both future research and practice related to reducing cancer risk disparities. An effective program targeting multiple cancer risk behaviors modeled after chronic care programs has the potential to make a large public health impact because of the dearth of evidence-based interventions for Latinos and the extended period of support that is provided in such a program.

**Trial registration:**

National Institutes of Health Clinical Trials Registry # NCT01504919

## Background

Over 60% of U.S. cancer mortality is attributable to tobacco use, poor nutrition, and physical inactivity [1,2], with almost one-third of all cancers directly attributable to tobacco use alone. Smoking cessation is associated with decreased risk of lung cancer, other cancers, heart attack, stroke, and chronic lung disease [3]. Diet accounts for 35% of cancer deaths, with diets low in fat, and high in fruits, vegetables, fiber, and grain associated with reduced risk [4-6]. A healthy diet is protective against lung, colon and rectum, breast, oral cavity, esophagus, stomach, pancreas, uterine cervix, and ovary cancer [7]. On the other side of the energy balance equation, regular physical activity can reduce the risk of colon and breast cancer [8-10]. Additionally, balancing "energy in/energy out" (i.e., calories eaten vs. expended) is imperative to avoid weight gain and the increased risk of cancer and other chronic diseases associated with overweight and obesity. Excess body weight has been found to be related to increased risk of as many as 14 types of cancer [11]. Furthermore, these cancer risk factors tend to be clustered, with smokers particularly likely to be physically inactive and have poor diets [12-14]. The presence of multiple cancer risk factors has synergistic adverse effects on health [15]. Thus, strategies for addressing cancer risk reduction among high-risk individuals need to address these multiple health risk behaviors.

The need for effective, culturally tailored behavior change interventions targeted at Latinos is critically important to public health for several reasons. First, Latinos, particularly of Mexican origin, are the fastest growing and largest minority group in the U.S. There are more than 40 million Latinos residing in the US, representing 14% of the total population [16], and people of Mexican origin account for 59% of this population [16]. Second, three of the four leading causes of death among Latinos cancer, heart disease, and stroke; [17] share smoking, poor diet, and physical inactivity as risk factors. Third, there are notable disparities in these risk factors for the Latino population. For example, although the prevalence of smoking is lower among Latinos than among the general population 16% versus 21%; [18], Latinos who smoke are less likely to receive advice to quit from a health professional, and to use cessation counseling services or medication compared to African American or White smokers [19]. National data indicate that 75% of Latinos do not eat the recommended servings of fruits and vegetables and 58% are physically inactive, with both Latino men and women being less likely to meet the recommended levels of fruits and vegetables and physical activity compared to non-Hispanic Whites [20]. Fourth, there is a strong clustering of these risk factors among Latinos and disparities exist in this clustering relative to other racial/ethnic groups. Latino smokers have both the highest prevalence of overweight/obesity (79%) and the highest number of additional risk factors compared to African American or White smokers [21]. Finally, 43% of Latinos speak Spanish at home and have limited English proficiency, whereas another 36% speak predominantly Spanish at home [16]. Thus, cancer prevention efforts among Latinos should include Spanish language programs if they hope to have a large public health impact.

Despite the critical importance of reducing cancer risk factors in Latinos, few stringent tests of targeted interventions have been conducted examining behavior change related to smoking cessation, diet or physical activity. For example, in contrast to the hundreds of smoking cessation treatment studies conducted among the general population [22], a recent review found only 12 studies that targeted Latino smokers [23]. Further, although all attended to cultural or linguistic tailoring to some extent, only five of those 12 studies utilized experimental designs. None of these studies attempted to simultaneously affect smoking and another health behavior. The situation is similar for diet and physical activity interventions among Latinos. A recent review found 16 culturally tailored interventions that examined behavioral outcomes (vs. knowledge/attitudes) and were tested with an experimental design [24]. Five of the 16 examined only diet, two examined only physical activity, and eight examined both. One study examined smoking and fruit/vegetable consumption. Five of the 12 smoking interventions and 12 of the 16 diet and physical activity interventions demonstrated significant effects. Thus, although notable efforts to create tailored or targeted interventions for Latinos have been made, there continues to be a dearth of evidence-based interventions for interventions targeting multiple cancer risk behaviors. Interventions addressing multiple cancer risk factors have shown great promise, and there is evidence that attempts to change one risk factor often leads to interest in modifying other risk factors, reflecting potential synergies in changing overall cancer risk profiles [25-28]. In sum, there is a compelling public health need to develop behavior change interventions for Latinos that address multiple cancer risk factors. Further, the development and evaluation of behavior change interventions for Latinos and other underserved groups has been identified as a national health priority [22,29].

### Study objective and aims

The goal of this randomized controlled trial is to evaluate the efficacy of a telephone-based counseling intervention that simultaneously addresses three cancer risk factors (smoking, fruit/vegetable consumption, physical activity) among overweight/obese Mexican-origin smokers in Houston, Texas. The intervention builds on previous work demonstrating the efficacy of the intervention for smoking cessation among Latinos of low socioeconomic status SES; [30], and among female smokers [31], and efficacy in preventing postpartum smoking relapse among a diverse sample of low SES women [32]. Additionally, the intervention is informed by data demonstrating the feasibility of reaching Spanish-speaking Latinos in Texas via a telephone-based intervention [30], and work demonstrating the efficacy of telephone-based interventions for diet and physical activity [33,34].

The specific aims of the study are to: 1) test the efficacy of Motivation and Problem Solving (MAPS) to promote and facilitate cancer risk reduction among high-risk Mexican-origin individuals (overweight/obese smokers). Relative to a Health Education (HE) condition, MAPS is hypothesized to result in positive changes in each of the primary smoking, diet, and physical activity outcomes, and; 2) assess the effects of MAPS on hypothesized treatment mechanisms (e.g., motivation, agency/self-efficacy, stress/affect) and their potential as treatment mediators.

### Motivation and problem solving (MAPS)

MAPS is a holistic, dynamic framework for behavior change that integrates treatment elements from both motivational interviewing (MI) [35,36] and social cognitive theory [37,38]. The overarching theoretical rationale for MAPS is the social cognitive model of behavior change [38,39]. Social cognitive theory posits that “High levels of both motivation and self-efficacy are important ingredients … an individual may fail to engage in a specific behavior despite high levels of self-efficacy if the motivation for performance is low or absent” [38]. That is, theory posits that effective behavior change treatments require both enhancing the motivation to achieve and maintain change, as well as developing the self-efficacy and skills necessary to do so. Similarly, Miller et al. [40] note that “the key element for lasting change is a motivational shift that instigates a decision and commitment to change. In the absence of such a shift, skill training is premature." Nevertheless, current interventions often focus largely on either motivation (e.g., MI-based approaches) or problem-solving/skills training (e.g., social cognitive approaches) despite the strong theoretical and empirical bases for focusing on both. When motivation is addressed, the focus is typically on motivating individuals to initiate behavior change, with little to no focus on the motivation to maintain change or recover from a relapse [22].

MAPS embeds empirically validated social cognitive approaches within an overarching motivational enhancement framework based on MI. MI is a directive but client-centered therapeutic approach designed to minimize resistance, enhance motivation for change, and increase self-efficacy in a non-confrontational manner [35,36]. Several meta-analyses have supported the efficacy of MI-based interventions for smoking, dietary behavior change, and physical activity [41-44]. MI has been found to be effective for promoting dietary change and physical activity, and our own research has demonstrated the efficacy of a motivational approach (MAPS) for smoking cessation [31,32] including among Spanish speaking Latino smokers [30]. Similarly, the social cognitive model has generated a tremendous amount of intervention research demonstrating that social cognitive treatments for smoking cessation, diet, and physical activity are effective [45,46]. However, the relative neglect of motivation reduces their ability to effect behavior change among individuals who are not motivated to change.

Further, although stage-based conceptualizations of behavior change emphasize both motivation and skills training, motivational shifts are conceptualized as relatively stable changes in “stage” [47]. In contrast, MAPS is relatively unique in that it conceptualizes motivation as a fluid construct that can fluctuate on a moment-to-moment basis depending on context. Counselors carefully assess and attend to changes in motivation so that treatment strategies are appropriately matched to motivation in the moment. MAPS utilizes a chronic care model (e.g., extended duration of treatment) and is built around a “wellness program” that in addition to focusing on cancer risk behaviors, also addresses life events, stressors, and other concerns (e.g., depression, family, financial, etc.). By addressing the larger context in which health behaviors occur, not only are many of the barriers for successful behavior change addressed, but adherence is increased because individuals perceive that the counselors care about them as whole people, and are not solely interested in their health behaviors.

### Major hypothesized mechanisms

Both research and theory identify motivation, agency, and stress/negative affect as critical mechanisms underlying behavior change [48-51]. As such, MAPS specifically targets these mechanisms and they are hypothesized to underlie MAPS effects on behavior change.

#### Motivation

A large body of evidence supports the role of motivation in the decision to change, the likelihood of change, and the maintenance of change. Motivation, measured in varying ways, predicts smoking quit attempts, smoking cessation success, dietary change, and change in physical activity [47,52-55]. There is also evidence demonstrating that motivation can change rapidly [56-58], consistent with models positing that motivation is dynamic and characterized by frequent fluctuations [58]. Motivation for the maintenance of change has received little attention despite the fact that social cognitive theory posits that “The final and most important stage of the change process is the maintenance stage. It is during the maintenance stage (which begins the moment after the initiation of abstinence or control) that the individual must work the hardest to maintain the commitment to change over time,” [38]. More specifically, the motivation for maintaining change may weaken and ambivalence may increase as the individual is exposed to temptations and stressors [38]. Therefore, MAPS includes a specific emphasis on motivation throughout the entire change process and on appropriate therapeutic responses to rapid fluctuations in motivation. In MAPS, the counselor continually attends to motivational cues and adjusts therapeutic strategies in response to even momentary changes in motivation.

#### Agency (sense of control, self-efficacy)

Human agency reflects the ability to intentionally affect one’s behavior or life situation. Agency is determined both by personal resources and by the contextual influences impinging on that individual [59]. Concepts encompassed under agency include sense of control and self-efficacy. Sense of control is a learned expectation that outcomes depend on personal choices and actions rather than on chance, other people, or forces outside one’s control [50,60]. Self-efficacy is a form of agency that is context and behavior dependent; i.e., self-efficacy varies based on the behavior to be performed and situational demands [50,51]. A greater sense of agency is reflected in greater self-efficacy when faced with situations that challenge one’s ability to initiate or maintain change. Self-efficacy is a consistent predictor of behavior change in smoking, physical activity, and fruit/vegetable consumption [61-66]. Therefore, based on both data and social cognitive theory [38] MAPS targets agency via the removal of barriers to change, standard problem-solving and coping skills training, and by increasing motivation [38,67,68].

#### Stress/negative affect

Stress and negative affect, measured in many different ways, are associated with behavior change [69-71]. In addition, the magnitude and trajectory of stress/negative affect over time are powerful predictors of change [69,70], as are individual differences in affective vulnerability (95–98). Thus, MAPS includes stress management and negative affect reduction strategies.

### MAPS adaptation

MAPS has demonstrated efficacy for smoking cessation among Spanish-speaking Latinos [30], to promote smoking quit attempts among women [31], and preventing postpartum relapse [32]. Thus, the MAPS program must be adapted to additionally address fruit/vegetable consumption and physical activity. Consideration of the needs and preferences of the Mexican-origin population in regards to fruit/vegetable consumption and physical activity must also be addressed during adaptation. Literature review, expert consultation, and focus groups with the target population will guide the adaptation.

#### Literature review

A review of the published literature will be conducted with the goal of identifying shared barriers and facilitators of behavior in regards to fruit/vegetable consumption, physical activity, and smoking cessation. The purpose of this is to identify potential targets of intervention common to all three cancer risk behaviors that can be addressed through the MAPS intervention. Literature reviews will also be conducted with the goal of identifying practical models of cultural self-awareness and cross-cultural communication skills to encourage culturally sensitive counselor-participant interactions.

#### Expert consultation

Two researchers (authors EGE and KR) who are experts in motivational interventions for diet and physical activity are being consulted for the current study. The purpose of this is to gain expert feedback on the incorporation of these target behaviors into the MAPS intervention, which heretofore has been tested only with regard to smoking [30-32].

#### Focus groups

Focus groups will be conducted to gain insights from the members of the target population on exercise and dietary habits, barriers, personal values, and the acceptability of program materials. Five focus groups will be conducted. Focus group participants must meet the same eligibility criteria as the randomized trial. A focus group guide is being developed with input from a subset of the study’s community advisory group that included questions about various elements of the interventions and materials. All recommendations for modifications to the interventions and materials will be considered.

### Pilot testing

Following MAPS adaptation, the intervention and study procedures will be pilot tested on up to 20 individuals. Pilot testing will replicate the study procedures through approximately 2–3 months post-baseline assessment. For evaluating the treatments, pilot procedures utilize the “technology model” developed by the Yale Psychotherapy Development Center [72]. Briefly, after developing a good working treatment protocol, the treatment is administered over a shortened timeframe to a small group of participants (typically about 5–7). The counselors and participants are extensively queried during this time as to the appropriateness, acceptability, usefulness, etc. of all materials, assessments, procedures, and counseling. Problems with the protocol usually become apparent fairly quickly and the protocol is then modified based on the feedback and a second round of pilot participants will be enrolled. After 2–3 cycles of pilot testing, the protocols are typically ready to be finalized. At that time, participants will be formally enrolled in the clinical trial. Pilot study participants will be compensated for assessments at a rate commensurate with the formal study participants.

## Methods/design

### Location and setting

The study site will be the Behavioral Research and Treatment Center of The University of Texas MD Anderson Cancer Center. All baseline assessments, outcome assessments, and HE sessions will occur at this location. Additionally, individuals in the MAPS condition will receive telephone counseling based out of the Department of Health Disparities Research at The University of Texas MD Anderson Cancer Center.

### Ethics and trial registration

The current study was funded by National Cancer Institute (NCI) Grant U54CA153505. The study was approved by the Institutional Review Board of The University of Texas MD Anderson Cancer Center (Protocol #: 2010–0606), and is registered on the National Institutes of Health Clinical Trials Registry (available at ClinicalTrials.gov. Registration #: NCT01504919).

### Study population and eligibility

The study population will be overweight or obese Spanish-speaking Mexican- or Mexican-origin smokers who reside in the Houston, Texas, metropolitan area. Inclusion criteria will be: 1) self-report of Mexican heritage; 2) age 18 or older; 3) current smoker with a history of smoking an average of at least one cigarette per day during the last year; 4) register an expired carbon monoxide level of at least five parts per million; 5) body mass index ≥ 25 based on measured height and weight; 6) ability to engage in low to moderate physical activity as determined by the Physical Activity Readiness Questionnaire PAR-Q; [73]; 7) blood pressure reading <140/90 millimeters of mercury (mm Hg); 8) speak Spanish, and; 9) have a valid home address and a functioning telephone number. Participants do not need to be motivated to change their behavior.

Participants with high blood pressure readings defined as ≥ 140/90 mm Hg; [74] and those deemed ineligible based on the PAR-Q will be able to participate if they provide a letter from a physician who will continue to monitor the participant during the research study. Exclusion criteria include: 1) women who are pregnant or currently lactating; 2) contraindication for nicotine patch; 3) other active substance abuse or dependence; 4) regular use of other tobacco products; 5) current use of tobacco cessation medications; 6) currently enrolled in another study; 6) another household member enrolled in the study, and; 6) a score below 38 on the Short Assessment of Health Literacy for Spanish Adults SAHLSA; [75]. Ineligible individuals will be given referrals to community resources.

### Recruitment and consent

Participants (N = 400) will be recruited from the ongoing Mexican American Cohort Study (n > 20,000) in the Houston area and from the community. The Mexican American Cohort study was created in 2001 to assess genetic and non-genetic risk factors for cancer in this population. Recruitment procedures have been described in detail elsewhere [76]. Briefly, participants have been recruited through multiple strategies, including random digit dialing, block walking in predominantly Mexican American neighborhoods (i.e., >75% based on 2000 U.S. Census), from community centers and health clinics, and networking through currently enrolled participants. Participants are contacted every 6 months to obtain updated health status and contact information.

For this study, overweight/obese smokers will be identified from the cohort study database as potential participants and will be contacted by research staff. Participants will additionally be recruited via radio and print advertisements and direct community outreach. Potential participants recruited via advertisements will be instructed to call the study telephone line and provide contact information to learn more about the study. All potential participants will be contacted via telephone by research staff to assess their interest in participating. Potential participants will receive a description of the study and will be asked if they are interested in participating. Those who are interested will complete screening over the phone (upon giving verbal consent to be screened), and a baseline visit will be scheduled within two weeks. At the baseline visit, a research coordinator will provide another detailed description of the study, answer questions, and obtain written informed consent to participate in the study. The entire consent process will be completed in Spanish by bilingual research staff, and consent documents will be written in Spanish.

### Study design

**Figure 1:**
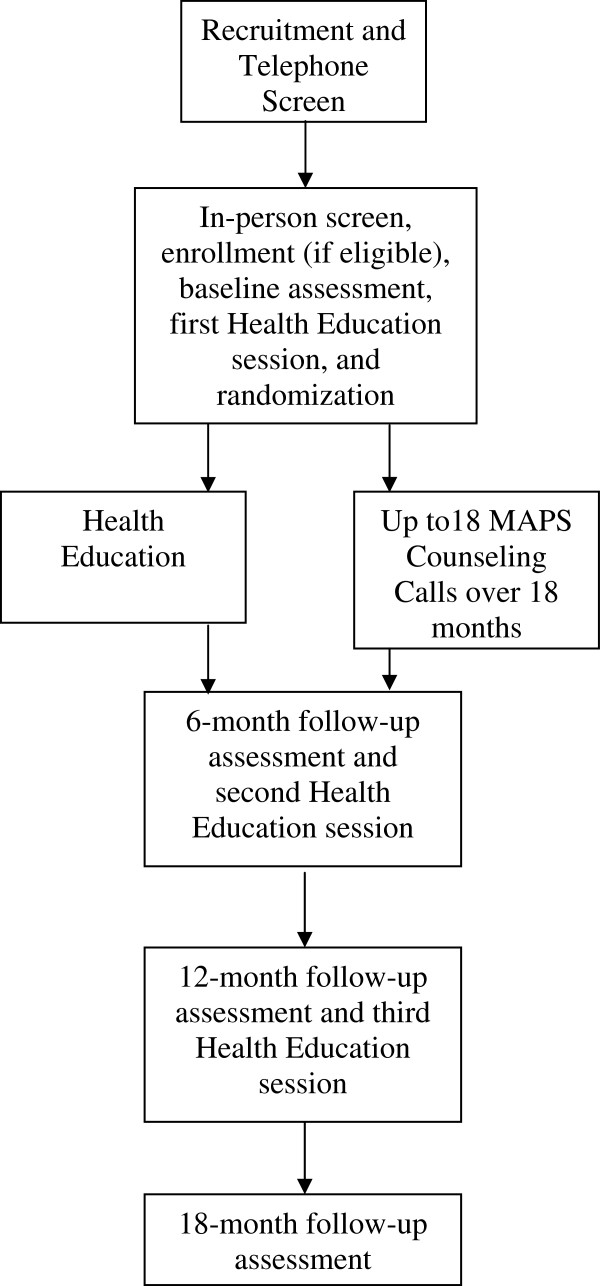
Study flow.

The current study is a two-group randomized controlled trial designed to test the efficacy of MAPS versus HE for multiple cancer risk behaviors among Spanish-speaking Mexican-origin smokers. Figure 1 depicts the study flow. Potential participants will call a phone number dedicated to study recruitment. They will undergo an initial eligibility screening (including BMI and smoking status eligibility based on self-report). Those who pass the initial screening and choose to participate will be scheduled to attend an in-person visit at the study site. Additional screening will take place, including objective verification of height, weight, and smoking status. Participants will also be screened for health literacy. After completing additional screening, those who choose to participate will complete the baseline assessment and the first of three HE sessions. After completing the first HE session, participants will be randomly assigned to HE or MAPS.

Randomization will occur using a form of adaptive randomization called minimization [77,78]. Compared to techniques such as stratification, minimization results in better group balance on participant characteristics. Minimization also provides for balanced treatment groups throughout the randomization process. Randomization will be balanced on gender, age, immigrant status, cigarettes per day, and BMI.

Individuals randomized to the MAPS condition will schedule their first counseling call during the baseline visit, and subsequently receive up to 18 counseling calls over an 18-month period. Outcome assessments will occur at baseline, 6 months, 12 months, and 18 months. Also after completing the assessment and randomization procedures, the 6-month follow-up visit will be scheduled for all participants. Research staff conducting the outcome assessments will be blinded to participants’ treatment conditions.

### Control and treatment conditions

#### HE condition

HE will include three sessions of brief advice (10 minutes) regarding smoking cessation, diet, and physical activity change, including the provision of referrals to resources for behavior change (e.g., Texas Quitline, community diet and physical activity programs). Participants will also receive Spanish language self-help materials. Participants will be given a home-based exercise kit (e.g., pedometer, exercise ball, strength training cables) and related instruction on the use of the exercise kit’s content during the first HE session. When the participant informs research staff that s/he is ready to make a smoking cessation attempt, a 6-week supply of free nicotine patches will be provided. HE will occur a total of 3 times (during the in-person assessment appointments at baseline, 6 months, and 12 months).

#### MAPS condition

The treatment condition will include HE plus up to 18 proactive, telephone-delivered MAPS counseling sessions over the 18-month period. Each counseling call will last approximately 20–30 minutes. Call frequency is negotiated between participant and counselor based on a participant’s motivation and needs. For example, several calls might be clustered around the initiation of a walking program, a smoking cessation attempt, holiday diet challenges, or specific barriers that the participant is facing (e.g., stress, lack of social support, family problems, financial crisis). Conversely, calls might be scheduled very infrequently when the participant is doing well. Regardless of when calls have been scheduled, participants are always able to call the study telephone line and request a counseling call.

All participants will complete a Wellness Plan in collaboration with their MAPS counselor. The Wellness Plan is an organization of goals or areas of concern/importance that the participant wants to work on during counseling. Wellness Plan items may include change goals for participants who are ready to make changes. The Wellness Plan may also include areas of concern or other topics that the participant would like to discuss where they may or may not be ready to change. Thus, the Wellness Plan will also be used in work with participants who are not motivated to make changes. This is because, although MI will be the primary task being used in working with these participants, it may be helpful to list areas of concern or topics that the participant is simply willing to explore and revisit. Wellness Plan goals can include not only goals related to the target behaviors, but also a plan for addressing other salient concerns such as stress, interpersonal issues, family problems, etc. The Wellness Plan can also include plans for connecting participants with resources in the community to address their needs, such as vocational and educational training, and free or low cost childcare and medical treatment. The Wellness Plan is a central organizational component of counseling. Thus, although a participant can develop a Wellness Plan as early as the first few sessions, it is revisited and revised over time to reflect participants’ progression through the program or any new areas of emphasis.

MAPS is designed to tailor counseling sessions completely around the participant’s expressed readiness to change a target behavior, and their unique needs and preferences for coping skills. Thus, it is unlikely any two participants will progress through the program at the same pace or express/address identical concerns. However, the general progression is broadly organized as follows: Early MAPS counseling sessions (e.g., 1–5) are intended for introduction to the target behaviors and an assessment of the participant’s motivation to change any or all of smoking, fruit/vegetable consumption, and physical activity. Where the participant expresses readiness, Problem Solving/Skills Training techniques are utilized to facilitate change. Where the participant is not yet ready, a MI approach is utilized. Later sessions (e.g., 7–16) are used either for continued MI in the areas where the client expresses not being ready to change, action oriented counseling to facilitate change, or maintenance of change, Sessions 17 and 18 prepare the participant for ending the program and include a final review of goals and progress, saying good-bye, and providing any additional necessary referrals.

#### Counselor qualifications, calls, and training

MAPS has been developed to be appropriate for delivery by lay and professional health workers. Counselors receive approximately 40 hours of MAPS training initially with “booster” sessions of approximately 1–2 hours every 1–2 months during the study. Training continues until the counselor reaches competence and adherence criteria. Miscellaneous counseling issues are also discussed at weekly project meetings. This level of training and ongoing monitoring is consistent with the recommended level of training for real-world providers of health promotion interventions such as Quitline counselors [79] and ensures that MAPS is of the highest quality and follows the protocol precisely, both of which are key fidelity issues in evaluating new treatments. Counselors will be two Spanish-speaking members of the study staff who are experienced in the delivery of MAPS.

##### Treatment fidelity

To monitor drift, all calls are digitally recorded and encrypted. A random sample of 10% of calls are coded using a modified Motivational Interviewing Treatment Integrity Manual MITI; [80]. The MITI has empirically validated reliability and validity, and is modified to include coding around appropriate social cognitive/problem-solving strategies, and transitions between the motivation building and problem-solving. Because MAPS utilizes a MI framework, the MITI works well for ensuring adherence to the protocol and utilizing the general MI spirit. A counselor who falls below performance criteria will receive additional training.

#### Instruments

The majority of the assessment instruments have been previously used and validated in Spanish, or are being tested in our current research. Assessment instruments measure outcomes or hypothesized mechanisms. We attempted to reduce the inconvenience associated with completing the assessment battery by providing financial compensation for participants’ time and by using the Questionnaire Design System (QDS), a computer-administered self-interview format. The error and time necessary to complete an assessment battery are substantially reduced by using QDS and particularly so among individuals with low literacy because the computer can read each item in Spanish to the participants. Assessment procedures will be identical at each of the four visits, with the exception that only a subset of the demographic measures will be administered at the 6, 12, and 18 month follow ups.

### Primary outcomes

#### Physical activity

##### International physical activity questionnaire (IPAQ)

The IPAQ assesses walking for exercise, walking for transportation, moderate and vigorous physical activity, and time spent sitting. It is widely used to measure physical activity [73].

##### Accelerometer

Participants will wear a small blinded accelerometer (i.e., participant cannot see values) over 7 days to assess physical activity. Accelerometers will be distributed at each assessment visit and will be returned via mail.

#### Fruit/vegetable consumption

##### National health interview survey diet items

Items will be used to assess frequency of food intake in 16 broad categories to estimate intakes of fruits and vegetables, percentage energy from fat, and fiber diets [81].

##### Two-item food frequency questionnaire

The two-item food-frequency questionnaire was developed to estimate intake of fruit and vegetables [82].

#### Smoking abstinence

Assessments will follow the recommendations from the Society for Research on Nicotine and Tobacco for cessation induction trials [83]. Participants will provide breath carbon monoxide to verify smoking. Although carbon monoxide has a relatively short half-life, the most comprehensive review on biochemical validation concluded that misreporting is typically very low (~2%) except for populations with substantial incentives to misreport (i.e., adolescents, pregnant women), and that adjustment for misreporting almost never influences analyses regarding relative treatment efficacy [84]. Participants will also provide a saliva sample to verify smoking status. The saliva sample will be provided at the baseline, 6 month, 12 month and 18 month follow-up visits. Saliva samples will be analyzed for cotinine, a metabolic byproduct of nicotine that provides an estimate of nicotine consumption. A saliva cotinine level of <20 ng/ml will be considered as abstinent.

#### Smoking status questionnaire

This surveys tobacco use, use of other tobacco products, and nicotine replacement medications. The questionnaire also collects data on the use of other tobacco products and nicotine replacement medications as determined by the participant's time point in the protocol (e.g., based upon the date the participant quits smoking) [83].

### Measures of hypothesized treatment mechanisms

#### Barriers self-efficacy physical activity

The Barriers Self-Efficacy Physical Activity is a 5-item measure on a five-point Likert-type and measures one’s confidence to meet a physical activity goal in the face of barriers to the behavior [85].

#### Fruits and vegetables self-efficacy

The Fruits and Vegetables Self-efficacy is a 7-item measure on a 5-Likert scales. Items assess family influences, decisional balances, and fruit and vegetable intake [85].

#### Fruits and vegetables staging

This is a 2-item measure of daily intake of fruits and vegetables and intent to change the current intake of fruit and vegetables.

#### Motivation

Motivation to abstain from smoking will be assessed with a 10-item scale currently being developed by our research team in order to assess motivation as a continuous variable as opposed to a stage. Items assess the motivation to quit smoking [86].

#### Positive and negative affect scale (PANAS)

The PANAS is comprised of two mood scales with high reliability, Positive Affect (PA) and Negative Affect (NA) [87].

#### Self-efficacy scale

This is a 9-item scale reflecting the confidence of the individual that they can cope with high-risk situations without smoking [88].

#### Smoking self-efficacy/confidence

The Smoking Self-Efficacy/Confidence scale assesses an individual's confidence to abstain from substance use or health behaviors in a variety of different situations. It consists of 9-item with 5 responses ranging from not at all confident to completely confident [88].

#### Smoking status questionnaire

This surveys tobacco use, use of other tobacco products, and nicotine replacement medications. The questionnaire also collects data on the use of other tobacco products and nicotine replacement medications as determined by the participant's time point in the protocol (e.g., based upon the date the participant quits smoking) [83].

#### Stages of change: physical activity

The Stages of Change: Physical Activity is a 1-item measure of the five stages of change in physical activity [89].

### Other measures

#### Brief Wisconsin Inventory of Dependence Motives

This questionnaire yields an overall dependence score as well as subscale scores for other critical dimensions of tobacco dependence (affiliative attachment, automaticity, loss of control, cognitive enhancement, craving, cue exposure/associative processes, social/environmental goads, taste, tolerance, weight control, and affective enhancement). The overall and subscale scores have high internal consistencies and predict abstinence [90].

*Demographics Questionnaire* collects data on age, race, ethnicity, education, income, preferred language, and generations in the U.S.

#### Medical history

Participants will be asked to provide a detail medical history including heart disease, asthma/lung disease, high blood pressure, diabetes, high cholesterol, thyroid problems, and kidney disease.

#### Medication worksheet

Participants will be asked to provide a detail list of medications and their dosage that they take daily.

#### Short assessment of health literacy for Spanish adults (SAHLSA)

The SAHLSA will be used to assess Spanish language health literacy. Participants are presented with a list of 50 common medical terms. Each word is presented with a synonym and a distracter word. The participant is instructed to pronounce each word and identify the synonym. A score of 38 is indicative of at least marginal health literacy, and higher scores indicate greater health literacy [75].

### Pharmacotherapy

All study participants will have access to free nicotine patch therapy when they are ready to make a quit attempt. The provision of patch therapy or other cessation medication is the recommended standard of care. As such, it is arguably an ethical obligation to provide all participants with access to patch therapy. Study participants receive nicotine patch therapy because the patch is currently recognized as a frontline therapy [22], and compared to non-nicotine medications such as bupropion or varenicline, the patch is safer, better tolerated, and available over the counter. Participants who decide to make a quit attempt will request patches by contacting project staff. Patches and instructions for their use will be distributed either via a visit to MD Anderson or via the mail. Patch therapy for participants who smoke >10 cigarettes/day will consist of 4 weeks of 21 mg patches, 1 week of 14 mg patches, and 1 week of 7 mg patches. Patch therapy for participants who smoke 6–10 cigarettes/day will consist of 4 weeks of 14 mg patches and 2 weeks of 7 mg patches. Patch therapy for participants who smoke 1–5 cigarettes/day will consist of 6 weeks of 7 mg patches. A reduction in dosage or cessation of the patch regimen will be implemented for any participants who report signs of being on too high of a dose, although this is typically not necessary for participants because blood nicotine levels are usually far lower on the patch than while smoking [91]. In sum, all participants, regardless of treatment group assignment, will have the same access to nicotine patch therapy. We will carefully track pharmacotherapy use at all follow-up assessments.

### Participant compensation and retention

To compensate for the time and inconvenience associated with participation, participants will be reimbursed $25 for each of the 4 assessments (i.e., up to a total of $100). Focus group participants will receive a gift card worth $50. Pilot study participants will receive a $25 gift card at each visit (Day 1 and Month 3), for a total of up to $50 in gift cards. Participants will not receive compensation for the counseling calls. Other procedures to reduce attrition include: 1) mailing postcard reminders and calling to remind participants of upcoming visits; 2) maintaining communication with participants throughout the study via birthday cards, holiday cards, etc. (each mailing includes a stamped address update postcard to update contact information); 3) having research staff member available during daytimes, evenings, and weekends to conduct study visits; 4) requiring that in addition to a functional phone number (necessary for counseling calls), participants have a home address so that they can be contacted by mail if necessary; and 5) obtaining names, addresses, and phone numbers of up to three collaterals (i.e., relatives/friends) who can provide information on participants’ whereabouts during the study (permission to contact the collaterals will be obtained from participants).

### Data analysis

Primary outcomes used to evaluate the efficacy of the MAPS intervention (Aim 1) are smoking status, servings of fruits and vegetables, and both self-reported and objectively measured physical activity assessed at the 6, 12, and 18 months. Because the primary outcomes and mechanisms include repeated measurements that are correlated within subjects, the data analytic approach utilizes generalized linear mixed model regression GLMM; [92,93]. Model diagnostics will be used to determine and address the form of the covariances, transformations, collinearity, and influential observations. GLMM parameter tests will be conducted using Wald statistics, and will be adjusted for multiple comparisons where appropriate. Adjustments for multiple comparisons will be made according to the method of Westfall and Young [94]. In this approach, the correlations among the dependent variables are used to adjust the critical values of the individual tests to ensure that the probability of a Type I error across a set of tests does not exceed the chosen alpha level. This approach has the advantage of maintaining the chosen Type I error rates while at the same time providing a less conservative adjustment than Bonferonni-type procedures.

#### Aim 1

Evaluate the efficacy of a MAPS approach to promoting and facilitating reduction of behavioral risk factors for cancer among high-risk Mexican-origin individuals (overweight/obese smokers). Relative to HE, MAPS is hypothesized to result in positive changes in each of the primary smoking, diet, and physical activity outcomes.

GLMM will be utilized in analyzing the effects of MAPS on the primary outcomes across the 6, 12, and 18-month time points. For the dichotomous outcome, we will assume a logit link and binomial variance function for the GLMM, and parameterize them with blocking on individual nested within treatment condition. Treatment and time will be included, as well as their interaction, with adjustment for relevant covariates as necessary. Time will be treated as a categorical variable. These models test for the main effects of treatment and time, and whether the treatment effect varies over time. For the continuous outcomes, the GLMM analysis will be replaced by linear mixed model (LMM) analysis (i.e., a special case of GLMM in which each outcome variable is continuous).

#### Aim 2

Assess the effects of MAPS on hypothesized treatment mechanisms (e.g., motivation, agency/self-efficacy, stress/affect) and the role of those mechanisms in mediating the effect of MAPS on outcomes.

To assess MAPS effects on treatment mechanisms, analyses largely analogous to the GLMM and LMM analyses described for Aim 1 will be conducted. Mediation will be indicated if: (a) there is a significant MAPS effect on the mechanism, and (b) the mechanism significantly predicts the outcome when adjusting for treatment. To formally test for the mediation effect, we will follow appropriate methods described by MacKinnon [95]. In particular, we will use a product-of-coefficients approach in which the indirect (mediation) effect is defined as the product of the coefficient of the intervention condition in the regression of the mechanism (*a* path) and the coefficient of the mechanism in the regression of the outcome variable controlling for intervention condition (*b* path), with appropriate coefficient standardization from *b* path, when the outcome is binary [95]. This approach is applicable for both continuous and binary outcomes (e.g., smoking status, servings of fruits and vegetables, and both self-reported and objectively measured physical activity), with and without repeated measurements. We will use a bootstrapping approach to computing confidence intervals of the indirect effects. Multiple mediator models will be fit to assess simultaneous mediation effects by multiple mechanisms. In addition to the tests for indirect effects, we will also calculate proportion mediated effects and standard errors in both the simple and multiple mediator models.

##### Missing data and drop-outs

Some individuals will fail to complete all assessments. GLMM is designed to handle missing data and will give valid inferences for effects provided that the probability of missing data depends only on the observed outcome and/or covariates in the model (or data are missing at random or missing completely at random). We will conduct analyses to examine whether participants who drop out of the study differ from those who do not, and control for those characteristics that are unbalanced between dropouts and completers and believed to be associated with the outcomes. In case where non-ignorable dropout [96] is suspected, our primary analysis approach will use a conservative one of coding the missing outcome as a failure. For example, a missing smoking outcome could be coded as smoking. In addition, we will conduct sensitivity analysis using selection models to account for non-ignorable dropouts. Specifically, we will follow the approach of Diggle and Kenward [97] to model the dropout as a function of both the currently unobserved and previously observed values of the outcome variables (e.g., when dropout is due to lack of improvement in the outcome). Alternatively, we can consider a slope-dependent dropout mechanism [98,99] to account for non-ignorable missingness. In this model, participants are believed to drop out with a high probability if the underlying (unobserved) rate of their change of outcome (e.g., physical activity level) over time is low. In spite of the availability of these potentially useful missing-data handling techniques, we do not expect they will lead to remarkably different results or conclusions given our consistently high follow-up rates [30,100-103].

### Power

All power analyses assume a significance level of 0.05 and a two-sided test and accounts for a potential 20% attrition at all time points (N = 320; 160/group). Three of the outcomes are continuous (servings of fruits and vegetables, METS of physical activity, accelerometer assessed activity). One outcome (smoking status) is dichotomous. Power is expected to be greater for intent-to-treat analyses than the power reported here, which represents completers only analyses. This is because the total number of cases for intent-to-treat analyses will be 400, versus 320 for the completers only analyses. For intent-to-treat analyses, those lost to follow up will be coded as not abstinent.

#### Continuous outcomes

Because treatment outcomes are measured at 6, 12, and 18 months, treatment effects may not be consistent across time points. In the case of a constant treatment effect across time, the detectable difference depends on the intraclass correlation coefficient (ICC) between measurements taken from a given participant. Since 160 participants per arm are assumed after attrition, and there are 3 measurements per participant, the effective sample size (ESS) per arm is 3*160/VIF, where VIF is the variance inflation factor 1 + (p-1)*ICC. In this formula, p is the average number of observations per participant. Table 1 shows the minimal detectable constant treatment effect under a range of values for the ICC when testing at a power of 80%. The detectable differences are given in terms of the standard deviation of the error term of the model. Thus, if the ICC is 0.3, the current study has 80% power to detect a shift of 0.229 standard deviations between the treatment arms, a small effect size per Cohen [104]. Power calculations displayed in Table 1 assume approximately consistent effects of treatment across measurements. However, correlations between more distal time points are generally lower than more proximal time points. Thus, calculations presented in Table 1 represent a worst-case scenario. We further computed power for detecting an average intervention difference of 0.3 when the effect varies across time points, specifically, with a shift of 0.1 at 6 months, 0.3 at 12 months, and 0.5 at 18 months. A Geisser-Greenhouse Corrected F Test was used for testing the intervention effect. The estimated power for detecting the treatment effect was 99%, 96%, and 91% for ICC values of 0.1, 0.3, and 0.5, respectively. It is important to note that the presented case of a 0.1, 0.3, and 0.5 increasing difference across time points produces exactly the same power as that of a 0.5, 0.3, and 0.1 decreasing difference across those time points.

**Table 1:** Minimal detectable difference with constant effect

**ICC**	**VIF**	**ESS**	**Detectable difference**
0.0	1.0	480	0.181
0.1	1.2	400	0.198
0.3	1.6	300	0.229
0.5	2.0	240	0.256
1.0	3.0	160	0.314

Note: ICC = intraclass correlation coefficient; VIF = Variance Inflation Factor; ESS = Estimated Sample Size.

#### Binary outcomes

National data suggest that approximately 5% of the general population of smokers quit each year [105]. However, smokers in HE receive a brief treatment based on national recommendations [22]. Therefore, we estimate that the abstinence rate for HE will be approximately 5%, 10%, and 15% across the three follow-ups (i.e., 10% quit rate per year). Power calculations for correlated binary outcomes are more complex than continuous outcomes, which require simulations. For example, it is not possible to have the same ICC between all pairwise observations. Therefore, the mean of all pairwise ICC values was used as the overall ICC and looked at three potential scenarios based on the simulation of 1000 trials. Using the estimated quit rate for HE of 5%, 10%, and 15% across the three follow-ups, Table 2 shows simulation results for constant, increasing, and decreasing differences between arms for each time point at ICC values of 0.1, 0.3, and 0.5. For cessation induction trials such as this one, the most likely scenario would be increasing differences over time given that 80-90% of smokers will not be motivated to quit at Baseline. Regardless, power is very good for detecting reasonable and meaningful differences between groups under realistic scenarios.

**Table 2:** Simulation results

	**Constant differences**				**Increasing differences**				**Decreasing differences**		
	**6 months**	**12 months**	**18 months**		**6 months**	**12 months**	**18 months**		**6 months**	**12 months**	**18 months**
**HE**	0.05	0.10	0.15	**HE**	0.05	0.10	0.15	**HE**	0.05	0.10	0.15
**MAPS**	0.15	0.20	0.25	**MAPS**	0.10	0.20	0.30	**MAPS**	0.20	0.20	0.20
**difference**	0.10	0.10	0.10	**difference**	0.05	0.10	0.15	**difference**	0.15	0.10	0.05
**ICC**	**power**	**95% CI**		**ICC**	**power**	**95% CI**		**ICC**	**power**	**95% CI**	
**0.10**	0.99	0.98**-**1.00		**0.10**	0.99	0.98	1.00	**0.10**	0.99	0.98**-**1.00	
**0.30**	0.94	0.93**-**0.95		**0.30**	0.95	0.94	0.96	**0.30**	0.98	0.97**-**0.99	
**0.50**	0.79	0.76**-**0.82		**0.50**	0.84	0.82	0.86	**0.50**	0.98	0.97**-**0.99	

Note: HE = Health Education; MAPS = Motivation And Problem Solving; ICC = intraclass correlation coefficient; CI = confidence interval.

## Discussion

Interventions addressing multiple cancer risk factors have shown great promise, [25-27], and a variety of approaches and settings have been utilized to address multiple cancer risk factors. For example, both worksite and health center based multiple risk factor interventions have shown efficacy with respect to improving diet and physical activity, but smoking cessation results have generally not been as positive [25,26,106-109]. Overall, multiple risk factor interventions provide an exceptionally promising and efficient means by which to facilitate behavior change and reduce cancer risk. Further, telephone-based interventions have demonstrated efficacy and broad reach as a method of of delivering smoking cessation, physical activity and diet/nutrition interventions [30-34]. The proposed study builds on this work using an intervention approach that has already been proven effective addressing smoking [30]. This intervention will be adapted, through literature review, expert consultation, and qualitative work, to simultaneously address smoking cessation, fruit/vegetable consumption, and physical activity, and to be sensitive to the needs of the target population. To the best of our knowledge, the current study will be among the first multiple risk factor intervention studies to focus on Mexican-origin individuals in general, and more specifically on overweight/obese Mexican-origin smokers, an extremely high risk group.

MAPS combines two widely utilized and empirically validated approaches [36,39] into a comprehensive, proactive, holistic approach that is specifically tailored to the motivational state, life concerns, and needs/preferences of the target population. MAPS is built around an individualized “wellness program” that is based on both successful chronic care models, patient navigation programs, and feedback from underserved communities. In addition to providing the necessary long-term support and strategies to initiate and maintain change, such an approach could potentially be more cost-effective than shorter interventions because of its effects on increasing the durability of treatment effects. Despite enthusiasm for more chronic care type approaches to behavior change, there have been few attempts to actually develop such programs. Moreover, telephone-based multiple risk factor counseling can both reduce the total number of risk factors, and be most effective among individuals with the greatest risk i.e., among individuals with larger numbers of risk factors; [26]. Further, treating cancer risk factors within a chronic care model, as MAPS does, has the potential to boost long-term success rates by improving the ability of an intervention to promote motivation, aid change attempts, prevent relapse, and encourage recycling among individuals who are unsuccessful [110-113].

## Competing interests

The authors declare that they have no competing interests.

## Authors’ contributions

DWW conceived of the study and drafted the manuscript; YC lead the intervention adaptation efforts and drafted the manuscript; MEF and LLS assisted with adaptation efforts; EGE and KR consulted on the study design and adaptation efforts; YL conducted the power analyses. All authors consulted on study design and approved the final manuscript.

## Pre-publication history

The pre-publication history for this paper can be accessed here:

http://www.biomedcentral.com/1471-2458/13/237/prepub
